# Leucorrhea and Its Treatment*Read at Palestine meeting, November, 1901, of East Texas Medico-Chirurgical Society.

**Published:** 1902-03

**Authors:** R. Y. Lacy

**Affiliations:** Pittsburg, Texas


For Texas Medical Journal.

### Leucorrhea and Its Treatment.*

BY DR. R. Y. LACY, PITTSBURG, TEXAS.

It is not by choice, but by assignment, that I make an assault upon the treatment of leucorrhea.

It is well known that leucorrhea is only a symptom, and not a disease. It may be due to either a specific or non-specific cause. It is my purpose in this paper to deal strictly with the latter variety, that which is found so often in the better class of females, both in the married and unmarried, and especially in the former, as it is this class that most often seeks treatment.

It is due to a multitude of causes, and is characterized by a discharge of a pale color, escaping from the female genital fissure, and is commonly termed by the laity "the whites."

The condition may result from any of the morbid processes that lead to a hyper-secretion from the genital mucous surface or from the glands opening upon them, whether the mucous membrane be injured or entire. There are several distinct varieties of leucorrhea, anatomically speaking. The vulva, vaginal, cervical, intrauterine, and tubal.

In the vulva variety, which occurs most commonly in the young, there is a glairy, viscid secretion that bathes the opposed pudenda and stiffens into a crust on the surface of the labia and thighs; this secretion is passed out generally by the glands of the mucous membrane of the vulva, but may come from the vestibula surface, and sometimes from the glands of Bartholin.

Second, the vaginal variety, is white in appearance, acid in reac-

---

*Read at Palestine meeting, November, 1901, of East Texas Medico-Chirurgical Society.

tion, and is due to a general secretion from the vaginal mucous membrane; it is sometimes yellowish in color, and contains pus cells and epithelium scales. The first is a simple catarrhal condition, the latter red granular spots covered over with membrane that has lost its epithelium; the catarrhal form is most common in the young married female; the latter about the menopause, or may occur earlier, and be complicated with some of the other forms of leucorrhea; this may be produced by sexual excesses as well as by specific causes, or by a pessary, or other foreign substances, or any condition, in fact, which interferes with the circulation of the pelvis.

Third, cervical leucorrhea, tenacious white discharge, resembling the white of an egg, alkaline in reaction; it is sometimes seen as it lies in the cervical canal as a greenish or yellowish or redish color; cilliated epithelial cells are found in numbers in the discharge. This is sometimes mixed with fatty cells, and a purulent looking fluid sometimes results. This is the most common variety, and affects mostly women who have been mothers and during the reproductive age. It is produced by foreign bodies, such as pessaries, excessive coition, and infected discharge from the body of the uterus; it may also be caused by specific virus or displacements of the organs.

The intra-uterine variety, the discharge is similar to the cervical in looks and reaction, but not so tenacious, being more fluid and often tinged with blood. This variety may be found at any period of life, but as an independent affection, is found almost exclusively in virgins and unmarried women; it is often associated with endocervical disease, and also found in women suffering from leucorrhea preceding and following the menstrual period. In women suffering from amenorrhœa, it is sometimes expelled with a gush at the usual menstrual period; this has its source in the interior of the uterus proper.

Some authorities state that there is a leucorrhea due to a diseased condition of the tubes, but this has never been definitely proven.

*Diagnosis*: A careful diagnosis should always be made, and the seat and cause of the discharge ascertained.

Discharge from the vagina is white in color and contains flakey masses; the cervical discharge is thick in consistancy, more tenacious, and may be colored and sometimes has a fetid odor.

Discharge from the body of the uterus is thin, may or may not have odor, and often contains blood cells and pus corpuscles, according to the cause of the discharge. It is often necessary to examine the fluid under the microscope to make a positive diagnosis of its source.

The M. M. of the vagina consists of basement membrane with stratified epithelium, and gonococci also often found; the cervix is lined with cylindrical epithelium which are ciliated; the interior of the uterus is lined by a single layer of prismatic cilliated epithelium.

*Treatment.* The treatment of leucorrhea is divided into constitutional and local; the constitutional treatment consists in building up the system, and as a general rule anemia is present, which should be overcome. Hare recommends the following prescription:

Acid arseniosi ......................gr. 1/4
Ferri redacti ......................grs. 5
Quinia sulph........................grs. 20
Ft. in pill No. 20. Sig.: 1 pill t. i. d. P. C. for adult.

Of the local forms of treatment, hot astringent douches for the vulva and vaginal forms have given the best results, in conjunction with the constitutional treatment above named.

In the cervical variety, the first step should be to correct the cause. If due to a displacement or laceration, this should be properly attended to, which will relieve the condition, and if of a catarrhal character, curettage and the application of equal parts of iodine and carbolic acid, and the prescription as stated to overcome the anemia, will usually effect a cure.

The same applies to the intra-uterine. The canal after curettage should be dried as thoroughly as possible, and a solution of iodine and carbolic acid introduced on a cotton wrapped applicator, if necessary, carrying it into the interior of the uterus and making the application cover the entire mucous membrane.

This is usually followed by a copious discharge containing shreds of mucous membrane, epithelium and blood cells; this discharge subsides in a few days.

Three applications, as above described, is usually sufficient; these applications should be made about a week apart.

